# Read Mapping and Transcript Assembly: A Scalable and High-Throughput Workflow for the Processing and Analysis of Ribonucleic Acid Sequencing Data

**DOI:** 10.3389/fgene.2019.01361

**Published:** 2020-01-24

**Authors:** Sateesh Peri, Sarah Roberts, Isabella R. Kreko, Lauren B. McHan, Alexandra Naron, Archana Ram, Rebecca L. Murphy, Eric Lyons, Brian D. Gregory, Upendra K. Devisetty, Andrew D. L. Nelson

**Affiliations:** ^1^ Genetics Graduate Interdisciplinary Group, University of Arizona, Tucson, AZ, United States; ^2^ CyVerse, University of Arizona, Tucson, AZ, United States; ^3^ LIVE-for-Plants Summer Research Program, School of Plant Sciences, University of Arizona, Tucson, AZ, United States; ^4^ Biology Department, Centenary College of Louisiana, Shreveport, LA, United States; ^5^ Department of Biology, University of Pennsylvania, Philadelphia, PA, United States; ^6^ Boyce Thompson Institute, Cornell University, Ithaca, NY, United States

**Keywords:** RNA-seq, transcriptomics, high throughput (-omics) techniques, bioinformatics, workflow

## Abstract

Next-generation RNA-sequencing is an incredibly powerful means of generating a snapshot of the transcriptomic state within a cell, tissue, or whole organism. As the questions addressed by RNA-sequencing (RNA-seq) become both more complex and greater in number, there is a need to simplify RNA-seq processing workflows, make them more efficient and interoperable, and capable of handling both large and small datasets. This is especially important for researchers who need to process hundreds to tens of thousands of RNA-seq datasets. To address these needs, we have developed a scalable, user-friendly, and easily deployable analysis suite called RMTA (Read Mapping, Transcript Assembly). RMTA can easily process thousands of RNA-seq datasets with features that include automated read quality analysis, filters for lowly expressed transcripts, and read counting for differential expression analysis. RMTA is containerized using Docker for easy deployment within any compute environment [cloud, local, or high-performance computing (HPC)] and is available as two apps in CyVerse's Discovery Environment, one for normal use and one specifically designed for introducing undergraduates and high school to RNA-seq analysis. For extremely large datasets (tens of thousands of FASTq files) we developed a high-throughput, scalable, and parallelized version of RMTA optimized for launching on the Open Science Grid (OSG) from within the Discovery Environment. OSG-RMTA allows users to utilize the Discovery Environment for data management, parallelization, and submitting jobs to OSG, and finally, employ the OSG for distributed, high throughput computing. Alternatively, OSG-RMTA can be run directly on the OSG through the command line. RMTA is designed to be useful for data scientists, of any skill level, interested in rapidly and reproducibly analyzing their large RNA-seq data sets.

## Introduction

**Figure 1:**
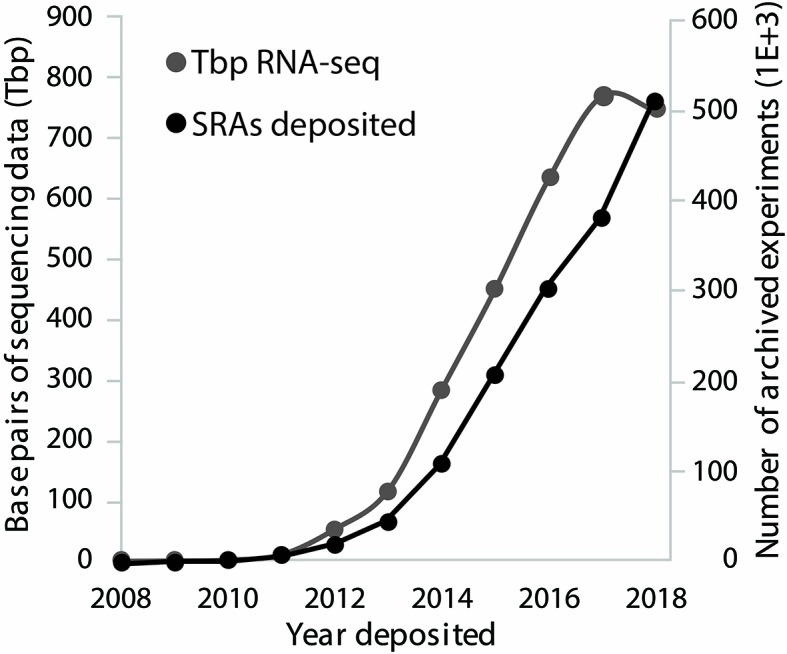
RNA-sequencing (RNA-seq) data deposited on National Center for Biotechnology Information (NCBI's) sequence read archive (SRA). SRA run information associated with transcriptomic analyses was downloaded and sorted by year deposited. Tera base pairs (Tbp, 1E+12) of RNA-seq data deposited is shown with the gray line and plotted on the left y-axis. Thousands of experiments deposited, per year, is shown with the black line on the right y-axis.

RNA-sequencing (RNA-seq) provides scientists with the ability to monitor genome-wide transcription across numerous cells or tissues and between experimental conditions in a rapid and affordable manner. Data generated from RNA-sequencing are incredibly powerful for differential gene expression analysis (Mortazavi et al., 2008; Li et al., 2016; Schlackow et al., 2017), novel gene discovery (Martin et al., 2013; Nelson et al., 2017), transcriptome-wide structural analysis (Gosai et al., 2015; Anderson et al., 2018), and even transcriptome-wide association studies (Galpaz et al., 2018; Gusev et al., 2019). In addition to generating and examining novel RNA-seq data, scientists are re-examining the hundreds of thousands of publicly available archived datasets to make novel discoveries (Lachmann et al., 2018), an analytical feat that represents a bottleneck for most researchers. The popularity of RNA-sequencing is perhaps most apparent by examining the dramatic increase in the number of RNA associated sequence read archives (SRAs) deposited in National Center for Biotechnology Information (NCBI's) SRA (Leinonen et al., 2011; **Figure 1**) over the last 10 years.

Alignment-based processing of these massive volumes of RNA-seq data typically involves two computationally intensive steps: mapping reads against a reference genome and transcript assembly. Reference genome based read mapping is performed using splice-aware algorithms such as STAR (Dobin et al., 2013) or HISAT2 (Pertea et al., 2016). The computational cost associated with mapping reads is dependent on the size of the genome and the number of reads to be mapped but typically takes hours to days on a standard lab server. The mapped reads are then used to assemble transcripts using programs such as StringTie or Cufflinks. Transcript assembly is less computationally intensive than read mapping but can still require several hours to complete. In addition to the computational requirements, both of these steps require substantial data storage resources and technical skills in transferring and manipulating large files, further increasing the technological burden for the researcher.

Successful assembly of RNA-seq data is insufficient to achieve the ultimate experimental goal: extraction of meaningful data. Data extraction usually involves differential expression analyses, isoform analysis, or novel gene identification. Each of these analyses requires different input file types and the use of different applications—each with their own intricacies surrounding installation, use, and preference for a Linux environment. In addition, preparing data files and then organizing them into the appropriate file structure for these next steps rapidly becomes tedious when performed on hundreds to thousands of files. Thus, despite the wealth of computing resources, extracting meaningful knowledge from RNA-seq data is still a non-trivial task.

Cloud-computing cyber-infrastructure platforms such as CyVerse (Merchant et al., 2016) and Galaxy (Afgan et al., 2016) have lifted the computational and data management burdens and made RNA-seq analysis more accessible to non-traditional data scientists. In contrast to fee-based services such as the Cancer Genomics Cloud (Lau et al., 2017) or FireCloud (Chet et al., 2017 – doi 10.1101/209494), CyVerse and Galaxy are free to users and provide long-term data storage solutions integrated with limited on-demand cloud compute resources. CyVerse and Galaxy also offer graphical user interface (GUI) platforms which allow researchers with minimal programming experience to easily deploy and handle large volumes of jobs in parallel. A complement to single-source resources like CyVerse and Galaxy is the Open Science Grid [OSG (Pordes et al., 2007)], a distributed computing resource capable of handling hundreds of thousands of jobs and transferring hundreds of petabytes of data per day. Thus, these computational resources make large dataset analysis and re-analysis feasible in a reasonable time-frame and cost-effective way.

Here we introduce RMTA (Read Mapping, Transcript Assembly), a high throughput RNA-seq read mapping and transcript assembly workflow. RMTA is easy to use and incorporates features that move beyond the standard RNA-seq workflow, allowing data scientists to focus their time on downstream analyses. For users with access and familiarity with high-performance computing (HPC) command-line operations, RMTA is packaged as a Docker container for one-step installation (**Table 1**). In contrast to other containerized RNA-seq analysis tools (Folarin et al., 2015; Jensen et al., 2018), RMTA is also installed as an app in CyVerse's Discovery Environment, which obviates computing and data storage requirements while providing a GUI for users less familiar with the command-line. Finally, for users querying extremely large data sets, OSG-RMTA marries the computational resources of the OSG with the job scheduling, data storage and management capabilities of CyVerse. Beyond read mapping and assembly, RMTA has a number of additional features that automate onerous data transformation and quality control steps, thus producing outputs that can be directly used for differential expression analysis or novel gene identification. In addition, the output from RMTA may be rapidly integrated in downstream transcriptomic data visualization platforms to help researchers extract meaningful knowledge. RMTA is both straightforward to install and use, and is meant to be used by both advanced and novice data scientists in their examination of their RNA-seq data.

**Table 1:** Deployment options for read mapping and transcript assembly (RMTA).

Platform	App Name	Size of Datasets That Can Be Handled	Data Storage Available	Genome Services Available
DE	RMTA v2.5.1.2	1–100	Yes	Yes
DE	OSG-RMTA v2.5.1.2	100–1000s	Yes	Yes
DE	RMTA-Instructional	1–10	Yes	Yes
Local	RMTA in Docker	Restricted to user capacity	No	No
OSG*	OSG-RMTA	100–1000s	No	No

Platforms include the Discovery Environment, a local computer, or high performance computing center, or the Open Science Grid. *Users wishing to utilize the Open Science Grid (OSG) outside of the Discovery Environment will need their own OSG account.

## Materials and Methods

In this section we provide an overview of RMTA, its different features, and its deployment options.

### Overview of the Read Mapping and Transcript Assembly Workflow

**Figure 2:**
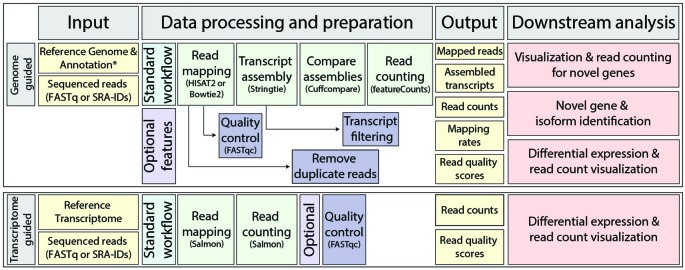
Read mapping and transcript assembly (RMTA) workflow with suggested downstream analyses. The standard RMTA workflow consists of read mapping by either HISAT2 or Bowtie 2, transcript assembly by StringTie, assembly comparison to the reference annotation by Cuffcompareto identify novel transcripts, and then read counting by featureCounts. Several optional features are included, such as the ability to perform quality control on RNA-sequencing (RNA-seq) data with FastQC, filtering of lowly expressed transcripts, and removal of duplicate reads (Bowtie 2 only). Output is listed, and are ready for downstream analyses such as those shown.

RMTA automates the three critical steps of RNA-seq analysis: read mapping, transcript assembly, and read counting. For genome-guided read mapping, RMTA utilizes either the splice-aware algorithm HISAT2 or the splice-unaware algorithm Bowtie 2 (Langmead and Salzberg, 2012) for mapping and then StringTie (Pertea et al., 2016) for transcript assembly (**Figure 2**). Minimum input requirements include a reference genome (FASTA or pre-indexed), and RNA-seq reads as either compressed or uncompressed FASTq, or as a list of one to thousands of SRA IDs. A reference genome annotation file (in GFF/GFF3/GTF) is optional and allows for downstream novel gene identification. RMTA automatically builds a reference genome index (if it is not provided) from the user supplied reference genome, aligns reads to the genome, and then returns a binary encoded version of a sorted sequence alignment map (BAM) file for each input FASTq/SRA. This BAM file is then automatically used as input for StringTie, where it, along with the reference genome annotation, is used to assemble transcripts. Following transcript assembly, each BAM file is processed by featureCounts (Liao et al., 2014) to determine how many reads map back to each gene/exon in the reference genome annotation file.

As an alternative to genome-guided read mapping and transcript assembly, RMTA also allows for read alignment directly to a transcriptome using the quasi-aligner and transcript abundance quantifier Salmon (Patro et al., 2017; Srivastava et al., 2019). Minimum input for Salmon includes a reference transcriptome (in FASTA format) and then RNA-seq reads (as above). Salmon maps reads to the provided transcript assembly and then counts the number of reads associated with each transcript, generating an output file (quant.sf) that can immediately be used for differential expression. The utilization of Salmon is only appropriate when the user is wanting to rapidly test for differential expression and cannot facilitate the identification of novel genes or data visualization in a genome browser.

OSG-RMTA utilizes a similar workflow to RMTA. The primary difference is how the user plans on launching jobs and providing the necessary input data to the OSG. When launched directly from within the OSG through a user's personal account, the user must provide access to all necessary data (e.g. genomes, RNA-seq data, etc). Thus, we recommend users submit jobs to the OSG through CyVerse's Discovery Environment. When jobs are submitted *via* the Discovery Environment, it automatically prepares the information needed to run the job and submits it to the OSG *via* HTCondor (Thain et al., 2005) and requires no OSG account (**Table 1**). Once the job is launched OSG-RMTA uses the information provided by the Discovery Environment to retrieve input files, process the data, and upload the results back to the Data Store, allowing the user to submit and walk away.

RMTA is also available for implementation on a HPC, a public cloud-based computing system (i.e., XSEDE or Atmosphere), or a local compute system. For local or cloud-based computing, a Dockerized version of RMTA identical to that used in the Discovery Environment is available for use inside a Docker command line environment. However, the user will need to direct Docker to the location of the input files and assign the required “flags” that are hidden when using RMTA in the Discovery Environment. More information on how to run the Docker version of RMTA on a Linux/personal computer (PC)/Mac operating system (OS) and a list of all available flags are available here (https://github.com/Evolinc/RMTA). Docker requires root privileges and thus is not available for HPC where users are denied super user do “sudo.” For HPC systems, Docker can be used alongside Singularity (Kurtzer, et al., 2017; instructions found here: https://sylabs.io/guides/3.4/user-guide/).

### Additional Read Mapping and Transcript Assembly Features

Several additional features have been included in the RMTA workflow to facilitate data discovery and quality control. For users wishing to call single nucleotide polymorphisms from their RNA-seq [or DNA-sequencing (DNA-seq)] data in a high throughput manner, the read aligner Bowtie 2 (Langmead and Salzberg, 2012) has been included as an optional aligner in the RMTA workflow. When the Bowtie option is selected, HISAT2 and StringTie are both removed from the workflow, but the additional option to remove duplicate reads (important for population level analyses) becomes available.

Poor quality RNA-seq reads, particularly at the 5' or 3' ends as a result of adaptor contamination or a drop in sequencing quality, can lead to a significant population of unmapped reads. To help the user identify issues resulting from poor read mapping rates, the quality control tool FastQC (Andrews, 2010) is available as an additional option in the RMTA workflow for both genome or transcriptome-guided read mapping approaches. FastQC provides the user with both an overview of potential issues with their data, as well as summary graphs highlighting issues such as per base sequence quality and Kmer content. Because FastQC works on read files in FASTq format, and we envision many users running RMTA directly on SRAs, FastQC has been placed downstream of read mapping (**Figure 2**). When the FastQC option has been selected, BAM files are converted back into FASTq with mapped and unmapped reads, along with their associated quality score, retained. This FASTq file is then used as input for FastQC, and then deleted afterward to reduce disk usage. If issues are detected at the 5' or 3' of sequencing reads, RMTA includes additional options for specifically trimming bases off of either end during the next analysis. Sequencing reads of overall poor quality will simply not be mapped and therefore do not need to be trimmed, but will still be highlighted in the FastQC results.

RMTA is also designed to aid in the identification of novel genes such as long non-coding RNAs from genome-guided transcriptome assemblies. To help the user remove transcript assembly artifacts that can arise from low expression, and therefore improve their attempts at novel gene identification, RMTA has two options for filtering lowly expressed transcripts. The user can decide to filter based on low expression [denoted as fragments per kilobase of transcript per million mapped read (FPKM)], low/incomplete read coverage (read per base), or use both filters in combination. We find that applying both filters (e.g., setting them both to one) helps to remove a large percentage of poorly assembled transcripts.

### Output From RMTA

The RMTA workflow produces a number of files that are designed to be immediately useful for downstream analyses such as differential expression, novel gene identification, and single-nucleotide polymorphism (SNP) discovery. Directly within the RMTA_Output folder the user will find the sorted BAM files and the filtered transcript assembly files (in GTF). The naming convention of these files reflects the SRA or FASTq from which they were derived (i.e., the input ID will be prepended to the output files). The filtered transcript assembly file is prepared for immediate use in the novel long non-coding RNA (lncRNA) identification package, Evolinc (Nelson et al., 2017), whereas the sorted BAM file is ready for import and visualization within a genome browser such as EPIC-CoGe (Nelson et al., 2018) or Integrative Genomics Viewer (IGV) (J. T. Robinson et al., 2011). The user will also find a “mapped.txt” file in the RMTA_Output folder, which contains information about alignment rates for each input FASTq/SRA. Within the RMTA_output folder is a subfolder labeled “Feature_counts” which contains a featureCounts summary.txt file and a tab-delimited file containing the number of reads assigned to each gene/exon for each of the RNA-seq data sets analyzed. If using the transcriptome-guided mapping approach (i.e., Salmon), a single quant.sf file will be generated that will contain the counts of all reads mapped to each transcript in each of the RNA-seq datasets processed. If the user selected the FastQC option, there will be a subfolder within the Output folder called “FastQC_out.” This folder will contain a FastQC.html file for each data set examined. Clicking on this file within the Discovery Environment will open up a new tab in the user's browser where all of FastQC's output information will be displayed. If the user chose Bowtie as the read aligner and “remove duplicate reads” as an additional option, then the RMTA_Output folder will only contain a sorted BAM file with duplicates removed for each SRA/FASTq input file, as well as a mapped.txt file. No additional files will be generated. A similar file/folder structure is generated no matter how an RMTA job has been launched (DE/OSG/HPC).

### Deployment Options

The different deployment options for RMTA and the benefits associated with each are summarized in **Table 1**. RMTA is freely available as an app (RMTA v2.6.3) within CyVerse's Discovery Environment (https://wiki.cyverse.org/wiki/display/DEapps/RMTA+v2.6.3). Running RMTA within the Discovery Environment allows the user to take advantage of CyVerse's simplified data management and storage options through the Data Store. In addition, integrated in the Discovery Environment are a number of virtual interactive computing environment (VICE) apps, such as the DESeq2 RStudio app, that allow users to examine their data start to finish completely in the cloud (https://learning.cyverse.org/projects/vice/en/latest/). OSG-RMTA (v2.6.3) is available as a separate app within the Discovery Environment. Although the OSG-RMTA app outwardly looks identical to RMTA, jobs are submitted to the OSG by CyVerse on behalf of the user, while also automating data management and transfer between the Data Store and OSG. RMTA is available as a Docker image https://hub.docker.com/r/evolinc/osg-rmta/ for easy installation in a command line environment (e.g. XSEDE or PC) where Docker is already installed or where the user has the necessary privileges to install Docker. Additionally, Docker can run within Singularity (Kurtzer et al., 2017), which enables launching RMTA within an HPC environment. Having RMTA packaged within a Docker container abrogates the need for installation of prerequisite software. For users with an OSG account and for whom a CyVerse account is unnecessary, OSG-RMTA is already present on the OSG as a Docker image for immediate use. A brief tutorial on how to use RMTA and OSG-RMTA in the command line and OSG, respectively, can be found in the README.md at (https://github.com/Evolinc/RMTA). Finally, a stripped down version of RMTA (few visible options) aimed at introducing undergraduates to the concepts of RNA-seq is also available in the Discovery Environment (RMTA_Instructional) with instructions at (https://wiki.cyverse.org/wiki/display/DEapps/RMTA_Instructional).

### Additional Discovery Environment-Specific Features to Simplify Ribonucleic Acid Sequencing Analysis

Although RMTA and OSG-RMTA are packaged as Docker images for use outside of CyVerse's Discovery Environment (e.g. OSG or an HPC), we highly recommend using the Discovery Environment integrated RMTA apps to take advantage of both the Discovery Environment's GUI and CyVerse's integrated Data Store. The Data Store makes data management relatively easy [drag ‘n' drop as opposed to shipping hard drives to Amazon Web Services (AWS) (Zhao et al., 2013)]. A number of up-to-date genomes are available in the community Data Store and the Discovery Environment has an application programming interface (API) that can acquire any of the 50,000 additional genomes from CoGe (Lyons et al., 2008) or public/private databases if needed. A Discovery Environment app has also been developed to retrieve GTF and BAM files from subdirectories generated for each SRA (File_Select v1.0) and place them into a single, user-specified folder, making data management even easier.

Researchers running OSG-RMTA in the Discovery Environment can take advantage of two features that facilitate a “divide and conquer” approach to job submission to the OSG. Long (> 1,000s) lists of SRAs can be divided up into smaller lists using the File_Split v1.0 app. The Discovery Environment's HT Analysis Path List file feature then uses these lists to parallelize their job submissions to the OSG. (https://wiki.cyverse.org/wiki/display/TUT/Parallel+execution,+DE+(Discovery+Environment)+style). Thus, a thousand SRAs can be processed in roughly the same time it would take to process 100. All of this happens with a few clicks of a button.

### Data and Software Availability

RMTA and OSG-RMTA are freely available to use as an app on CyVerse's Discovery Environment or on the Open Science Grid (https://hackmd.io/s/rJjrqyAAQ). Detailed instructions on how to use RMTA in the Discovery Environment can be found at (https://wiki.cyverse.org/wiki/display/DEapps/RMTA+v2.6.3). Working within the Discovery Environment requires a modern hypertext markup language 5 (HTML5) capable browser and a free CyVerse user account (user.cyverse.org). Users wishing to use OSG-RMTA on the OSG directly (not through the Discovery Environment) will need an account (http://osgconnect.net/). The source code of the workflow is available at https://github.com/Evolinc/RMTA and https://github.com/Evolinc/OSG-RMTA and the Docker images for users wishing to adapt RMTA to novel environments are available at https://hub.docker.com/r/evolinc/rmta and https://hub.docker.com/r/evolinc/osg-rmta/. Test data for RMTA are present in the Discovery Environment and on GitHub.

### Data Visualization in EPIC-CoGe, Long Non-Coding Ribonucleic Acid Identification With Evolinc, and Analysis of Gene Expression

Two sorted.bam files, SRR2240264 (flower) and SRR2240265 (root) from an RMTA run on 100 paired-end (PE) SRAs were uploaded to EPIC-CoGe from CyVerse's Data Store using the LoadExp+ tool (Grover et al., 2017) in CoGe. Expression data were associated with the *Arabidopsis thaliana* (Col-0) genome (v10.02, id 16911). These two datasets are publicly available in the RMTA folder (id 2568) at www.genomevolution.org. To identify lncRNAs, all 100 “filtered.gtf” files from the 100 PE RMTA analyses were added to an HTPathlist file in the Discovery Environment. This HTPathlist file was then used as the input for a single Evolinc analysis in the Discovery Environment (Evolinc v1.7.5; Nelson et al., 2017). The updated annotation file from each Evolinc job were merged using the Evolinc_merge app (v1.0). FASTA sequence for all identified lncRNAs were extracted using the gffread utility in the Cufflinks package. GC content and length of all *Arabidopsis* protein-coding genes and the newly identified lncRNAs were calculated using a custom Perl script (**File S1**). Principal component analyses were generated in R (code in **File S2**) using the read count data from RMTA.

## Results

To demonstrate the utility of RMTA, we used our workflow (**Figure 2**) to process 1,000 *A. thaliana* SRAs (single-end reads) and 100 SRAs (paired-end reads) directly from NCBI's data repository, representing 1.27 terabases of RNA-seq data (**Table 2** and **Table S1**). SRA IDs were obtained from NCBI's SRA by searching the term “*Arabidopsis thaliana*” and then exporting all summary results to a tab-delimited file using NCBI's “Send to” API. For downstream analysis of the PE data, specific RNA-seq data from root (*n* = 68) and flower (*n* = 32) tissue were chosen from these summary results (**Table S1**). PE and single-end (SE) SRA IDs were copied into new list files in the Discovery Environment, partitioned into lists of 10 and 100, respectively, using File_Split-1.0. These 10 list files were then added to an HT Analysis Path List that subsequently became the input for the RMTA app. Two analyses were launched in the Discovery Environment (one for PE and one for SE) whereupon they were automatically divided up and submitted simultaneously as 10 jobs each. Specific options selected for these analyses were: HISAT2 for the aligner, a FPKM, and coverage cut-off threshold of 1, and Run FastQC selected. All other options were left as default.

**Table 2:** Mapping rates and time to completion for the example read mapping and transcript assembly (RMTA) analyses.

	Mapping rates					
	> 90%	90–75%	75–50%	<50%	Gbp mapped	Mbp/minute
**SE samples**	63%	16%	9%	12%	863	45
**PE samples**	76%	15%	5%	4%	406	26

RMTA was used to process 100 paired-end (PE) and 1,000 single-end (SE) *Arabidopsis* sequence read archives (SRAs). The percentage of these SRAs with mapping rates >90%, 90–75%, etc., are shown. Gbp = 1x10^9^ base pairs mapped. Mbp/minute = million base pairs mapped per minute.

Mapping rates and time to completion are shown in **Table 2** and **Table S1**. While the mapping rates for most (76%) of the PE SRAs were >90% (avg = 92.7%, **Table 2**), six SRAs displayed rates <75%. FastQC results were interrogated to identify potential reasons for why these mapping rates might be low and if 5' or 3' trimming of reads might facilitate better mapping. FastQC results revealed a significant enrichment of adaptor sequence for these samples. A subsequent relaunching of RMTA with 15 nts trimmed from the 5' end (an option within RMTA) resulted in improved mapping rates for all six samples. This demonstrates the utility of being able to analyze hundreds of SRAs at once with default settings, and then follow up with adjusted parameters for problematic samples.

**Figure 3:**
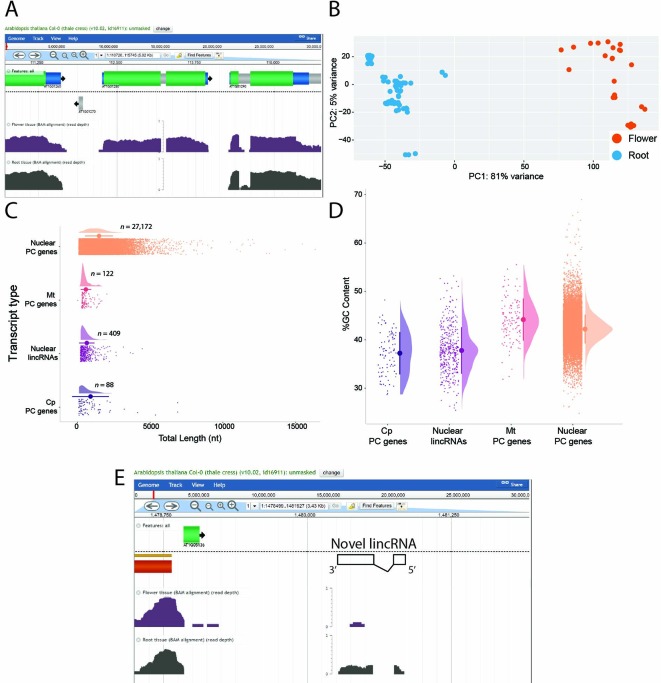
Examples of downstream analyses facilitated by the read mapping and transcript assembly (RMTA) workflow. The output generated by RMTA are immediately useful for the usual analyses performed following an RNA-sequencing experiment. **(A)** EPIC-CoGe screenshot of *Arabidopsis* root and flower RNA-sequencing (RNA-seq) data processed by RMTA highlighting a gene, *AT1G01280*, that is highly expressed in flower tissue but not roots. **(B)** Principal component analysis (PCA) of 100 *Arabidopsis* sequence read archives (SRAs) generated in R using the read count output file from RMTA. **(C)** Comparison of the length of Evolinc identified long non-coding RNA (lncRNAs) relative to other nuclear and organellar genes. PC, protein-coding gene; Mt, mitochondria; Cp, chloroplast. **(D)** Comparison of GC content of Evolinc identified lncRNAs relative to other nuclear and organellar genes. **(E)** EPIC-CoGe visualization of the expression of a locus identified by Evolinc as a lncRNA. The boundaries of the lncRNA and its orientation have been added to the EPIC-CoGe screenshot.

We then demonstrated three ways in which RMTA can facilitate downstream analysis: 1) by visualizing the RMTA generated BAM files in the EPIC-CoGe genome browser, 2) by testing for variation between datasets using the RMTA featureCounts output, and 3) identifying novel lncRNAs using RMTA's filtered genome annotation output. Users often wish to sanity check their RNA-seq data by viewing them in a genome browser. A benefit of performing RMTA in CyVerse's Discovery Environment is the ability to immediately import the large mapped read (BAM) files from the Data Store into the EPIC-CoGe genome browser (Nelson et al., 2018). Genomes for over 19,000 organisms are available on CoGe, meaning that the user will not only be able to visualize their RNA-seq, but can also import genomes from CoGe into the Discovery Environment to supplement the genomes already available. Two of the *Arabidopsis* PE-SRAs were imported into EPIC-CoGe (publicly available in the CoGe folder “RMTA,” ID: 2568) for public browsing (**Figure 3A**). For users performing RMTA locally (i.e., in a Docker container), genome browsers such as IGV (J. T. Robinson et al., 2011) are freely available and easy to use.

Sample variation within the 100 *Arabidopsis* PE-SRAs, consisting of RNA-seq data from 68 root and 32 flower samples (see **Table S1** for IDs) was examined using the RMTA-produced table of exon associated read counts (feature_counts.txt). While these analyses can occur using Discovery Environment RStudio VICE app deployments of DESeq2 (Love et al., 2014) or EdgeR (M. D. Robinson et al., 2010); https://learning.cyverse.org/projects/vice/en/latest/user_guide/quick-rstudio.html), as the output file from featureCounts is small and manageable, it was downloaded and manipulated in a local R environment (i.e., RStudio; RStudio Team, 2015; R-code available in **File S2**). A principal component analysis (PCA) demonstrated that, as expected, the largest amount of variation between samples (PC1) could be explained by tissue (**Figure 3B**). This analysis demonstrates the ease with which researchers can validate their RNA-seq data and proceed to differential expression analyses using the RMTA workflow.

The filtered genome annotation produced by RMTA is perfect for novel gene identification without any additional data transformation. To describe this, the RMTA output file “filtered.gtf,” with transcripts with an FPKM or read/base <3 removed, was used as input in the long non-coding RNA identification pipeline, Evolinc (v1.7.5; Nelson et al., 2017) in the Discovery Environment. Like RMTA, Evolinc is also packaged as a Docker image for local discovery. Evolinc was used to identify putative lncRNAs expressed in the root and flower RNA-seq data. The number of lncRNAs identified and some basic characteristics, such as average length and GC content relative to nuclear and organellar protein-coding genes, are shown in **Figures 3C, D**, with the R code necessary to recapitulate the images available in **File S3**. Reads mapped to these lncRNAs, and other novel genes, can also be visualized in a genome browser using the BAM files generated by RMTA (**Figure 3E**). In sum, RMTA is not only a simple and intuitive means of processing large amounts of RNA-seq data, but also facilitates commonly performed downstream analyses.

## Discussion

As the technical and financial barriers to generating raw RNA-seq data are reduced, the barrier to discovery will be shifted toward the computational steps required to analyze those data and the integration with other software for extracting high-value knowledge and novel scientific insights. RMTA was designed with the goal of alleviating many of the tedious or time consuming steps of RNA-seq processing and downstream data analysis. This goal was primarily accomplished by incorporating the three main steps of RNA-seq processing (read mapping, assembly, and counting) into a very approachable, yet scalable and interoperable tool, and ensure that the output files from RMTA are easily ingested by other platforms and analysis tools.

The usefulness of RMTA is most apparent when utilized within CyVerse's Discovery Environment. Access to public or private genomes (through CoGe and the Data Store), automatic data retrieval from NCBI's SRA, data management through the Data Store, and job submission within the Discovery Environment or direct to the Open Science Grid, means that data scientists can perform all of their analyses in the cloud. In addition, users can take advantage of parallelizable job submission options that are available to divide and conquer their large datasets. Once finished, RMTA produces output files that are ready for immediate analysis (e.g., differential expression), visualization (e.g., in a genome browser), or novel gene identification (e.g., long non-coding RNAs), all of which can also occur in the cloud. In sum, large-scale RNA-seq analysis is no longer limited to data scientists with HPC access or a high-end local computer.

RMTA was also designed for users who prefer to perform analyses locally. By packaging RMTA in a Docker container we have removed the tedious task of installing prerequisite software and made RMTA capable of running on any operating system. Thus, processing and analysis of RNA-seq data is no longer restricted to a Linux machine but can now also be performed on a machine utilizing Windows or Mac OS. In addition, recognizing data storage limitations, RMTA removes unnecessary files generated during the analysis that would rapidly fill up most storage allotments.

Not all data scientists have the same needs in terms of available features or in the amount of data to be processed. To this end we developed variants of RMTA targeting undergraduate or high school instructors (RMTA_instructional), users processing 1–100s of data files (RMTA), and users processing 1,000s or more data files (OSG-RMTA). RMTA_instructional is available as an app in the Discovery Environment with minimal fields exposed, with example input files added to the appropriate fields, and with entry level descriptions of the purpose behind each field. RMTA and OSG-RMTA are available as both Discovery Environment apps and Docker images, with OSG-RMTA already available on the OSG. RMTA and OSG-RMTA offer the same features, differing only in where and how jobs are submitted.

In summary, RMTA opens up the task of RNA-seq processing and data analysis to anyone with access to a web browser, thereby democratizing data discovery. It also enables analysis of all transcripts, not just the ones matching already annotated genes, thus encouraging a more inclusive view of what genomic regions are actually transcribed. Finally, RMTA serves as a useful tool for savvy data scientists wishing to reduce the time and effort necessary to process large data sets.

## Data Availability Statement

Publicly available datasets were analyzed in this study. Accession numbers can be found here: **Table S1**.

## Author Contributions

ADN, UD, EL, and BG designed the workflow. ADN, UD and SP wrote the code. SR and UD integrated RMTA into the OSG. SP, IK, LM, AN, AR, and RM tested RMTA, resolved bugs, and wrote the tutorials. SP, IK, LM, AN, AR, and RM analyzed the data. SP, RM, EL, and ADN wrote the manuscript. All authors read and approved the manuscript.

## Funding

This work has been supported by the National Science Foundation grants IOS—1758532 (to ADN, RM, and UD), IOS—1743442 (to CyVerse), IOS—1444490 (to EL and BG), NSF Research Experience for Undergraduates (REU to IK, LM, and AN), and an NSF Research Assistantship for High School Students (RAHSS to AR). As AR is a minor, parental consent has been given to include her as an author on this manuscript.

## Conflict of Interest

The authors declare that the research was conducted in the absence of any commercial or financial relationships that could be construed as a potential conflict of interest.

## Supplementary Material

Table S1 SRA IDs, tissue information, and mapping rates of example data.

File S1 Script used to calculate transcript length and GC content.

File S2 R code necessary to recapitulate PCA of example PE data.

File S3 R code necessary to recapitulate visualization of data.
