# Supplementary material for: Read Mapping and Transcript Assembly: A Scalable and High-Throughput Workflow for the Processing and Analysis of Ribonucleic Acid Sequencing Data
Source: Front Genet. 2020 Jan 24;10:1361. doi: 10.3389/fgene.2019.01361 (PMC6993073; doi:10.3389/fgene.2019.01361)
Supplement: Table S1 — SRA IDs, tissue information, and mapping rates of example data. [file DataSheet_1.pdf]

linc_RNA_violin

R. Murphy

6/27/2019

```r
#load libraries
#install.packages('Hmisc')
#install.packages('viridis')
library(readr)
library(ggplot2)
```

```
## Registered S3 methods overwritten by 'ggplot2':
##   method         from
##   [.quosures     rlang
##   c.quosures     rlang
##   print.quosures rlang
```

```r
library(Hmisc)
```

```
## Loading required package: lattice

## Loading required package: survival

## Loading required package: Formula

##
## Attaching package: 'Hmisc'

## The following objects are masked from 'package:base':
##
##     format.pval, units
```

```r
library(RColorBrewer)
library(viridis)
```

```
## Loading required package: viridisLite
```

```r
library(dplyr)
```

```
##
## Attaching package: 'dplyr'

## The following objects are masked from 'package:Hmisc':
##
##     src, summarize

## The following objects are masked from 'package:stats':
##
##     filter, lag
```

```
## The following objects are masked from 'package:base':
##
##     intersect, setdiff, setequal, union
```

```r
#setwd("C:/Users/moonb/OneDrive/Documents/R")

data <- read.csv(file = "For_becki_to_plot_3.csv", check.names=F, fileEncoding="UTF-8-BOM", stringsAsFa

head(data)
```

```
##   GC_Content Total_Length molecule
## 1   30.18868          212  lincRNA
## 2   39.59108          538  lincRNA
## 3   37.18245          866  lincRNA
## 4   28.90071         1128  lincRNA
## 5   38.19951          411  lincRNA
## 6   42.17391          230  lincRNA
```

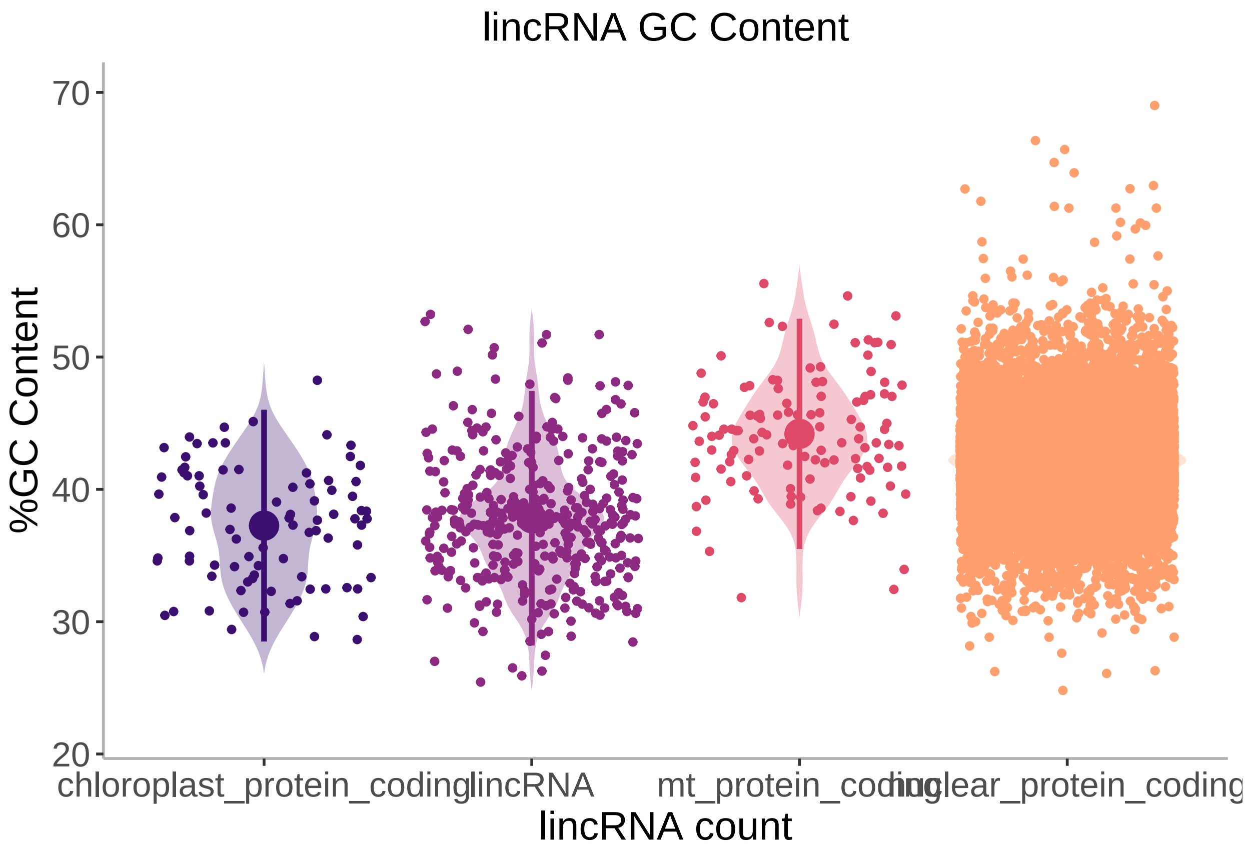


```
## Warning: Ignoring unknown parameters: se
```

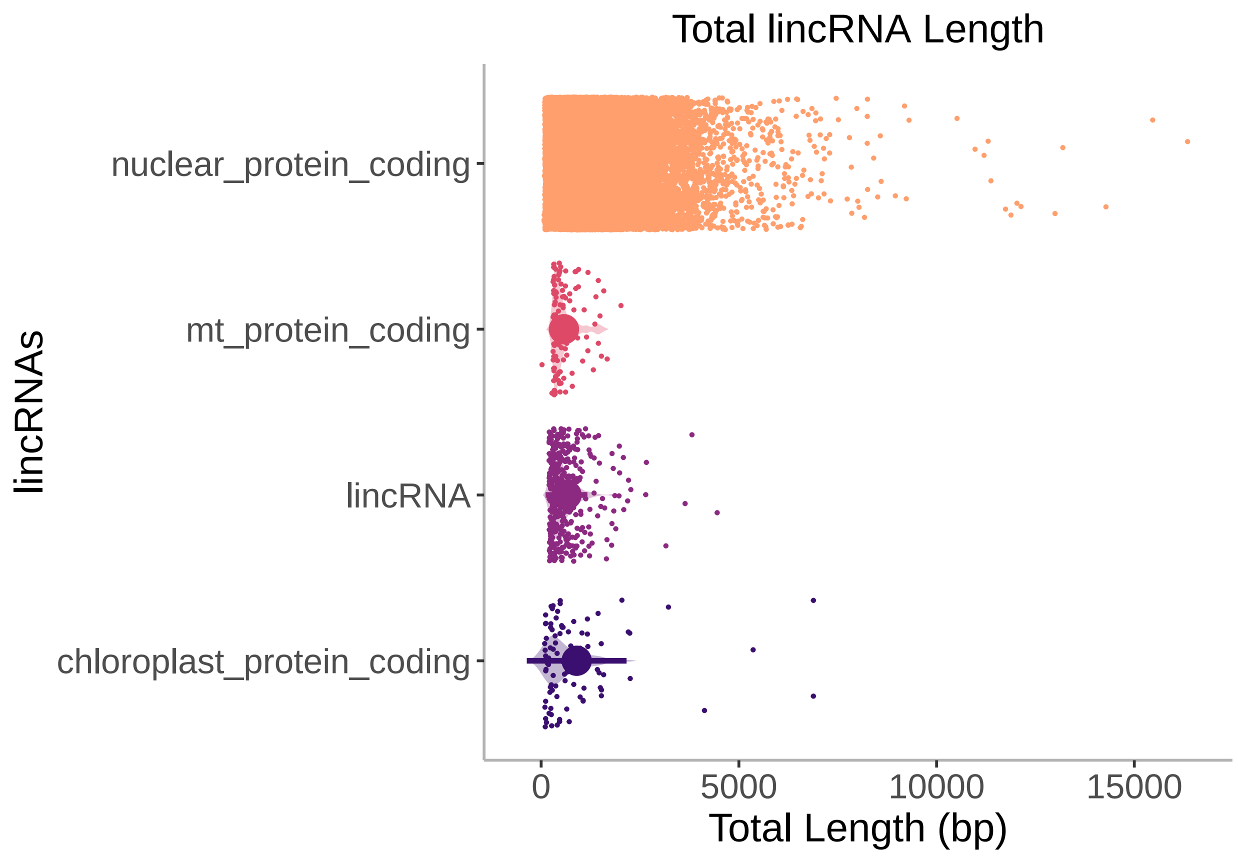


```
#setting geom_flat_violin as a function

"%||%" <- function(a, b) {
  if (!is.null(a)) a else b
}

geom_flat_violin <- function(mapping = NULL, data = NULL, stat = "ydensity",
                        position = "dodge", trim = TRUE, scale = "area",
                        show.legend = NA, inherit.aes = TRUE, ...) {
  layer(
    data = data,
    mapping = mapping,
    stat = stat,
    geom = GeomFlatViolin,
    position = position,
    show.legend = show.legend,
    inherit.aes = inherit.aes,
    params = list(
      trim = trim,
      scale = scale,
      ...
    )
  )
}

#' @rdname ggplot2-ggproto
```

```r
#' @format NULL
#' @usage NULL
#' @export
GeomFlatViolin <-
  ggproto("GeomFlatViolin", Geom,
          setup_data = function(data, params) {
            data$width <- data$width %||%
              params$width %||% (resolution(data$x, FALSE) * 0.9)

            # ymin, ymax, xmin, and xmax define the bounding rectangle for each group
            data %>%
              group_by(group) %>%
              mutate(ymin = min(y),
                     ymax = max(y),
                     xmin = x,
                     xmax = x + width / 2)

          },

          draw_group = function(data, panel_scales, coord) {
            # Find the points for the line to go all the way around
            data <- transform(data, xminv = x,
                              xmaxv = x + violinwidth * (xmax - x))

            # Make sure it's sorted properly to draw the outline
            newdata <- rbind(plyr::arrange(transform(data, x = xminv), y),
                             plyr::arrange(transform(data, x = xmaxv), -y))

            # Close the polygon: set first and last point the same
            # Needed for coord_polar and such
            newdata <- rbind(newdata, newdata[1,])

            ggplot2:::ggname("geom_flat_violin", GeomPolygon$draw_panel(newdata, panel_scales, coord))
          },

          draw_key = draw_key_polygon,

          default_aes = aes(weight = 1, colour = "grey20", fill = "white", size = 0.5,
                            alpha = NA, linetype = "solid"),

          required_aes = c("x", "y")
)
```

```r
      #plot for GC content




     gc_content_rain <- ggplot(data, aes(molecule, GC_Content, fill=molecule, color=molecule)) +
geom_flat_violin(position = position_nudge(x = .2, y = 0), alpha = .5, color='white') +
  geom_point(position = position_jitter(width = .15), size = .5, alpha = 0.8) +
 stat_summary(fun.data = "mean_sdl", fun.args = list(mult = 1), geom="pointrange", position = position_r
expand_limits(x = 5.25) +
```

```r
guides(fill = FALSE) +
guides(color = FALSE) +



  #data colors

  scale_fill_viridis(option = 'magma', discrete = TRUE, alpha = 0.3, begin = 0.2, end = 0.8) +
  scale_color_viridis(option = 'magma', discrete = TRUE, alpha = 1, begin = 0.2, end = 0.8) +



  # labels for the plots

  (labs(title = "A Comparison of GC Content", x = "lincRNA count", y = "%GC Content")) +


  #general aesthetics


  theme(

    plot.title = element_text(hjust = 0.5, size = 15),
    panel.background = element_rect(fill = 'white'),
    axis.title.y = element_text(size = 15),
    axis.title.x = element_blank(),
    axis.text.y = element_text(size = 13),
    axis.text.x = element_text(size = 13),


  #plot area and gridlines

    axis.line = element_line(size=0.5, colour="gray70"),
    panel.border = element_blank(),
    panel.grid.major.y = element_blank(),
    panel.grid.major.x = element_blank(),
    panel.grid.minor = element_blank(),

  #legend off

   #legends





  )


#plot for GC Content


print(gc_content_rain)
```
