# Supplementary material for: Read Mapping and Transcript Assembly: A Scalable and High-Throughput Workflow for the Processing and Analysis of Ribonucleic Acid Sequencing Data
Source: Front Genet. 2020 Jan 24;10:1361. doi: 10.3389/fgene.2019.01361 (PMC6993073; doi:10.3389/fgene.2019.01361)
Supplement: File S2 — R code necessary to recapitulate PCA of example PE data. [file Table_2.docx]

#!/usr/bin/perl -w

#Many thanks to the original author of this script (see below)

#To run, copy and save into a new file as a perl file (***.pl). Then follow usage parameters below

####################################################################################################

### Get GC Content ###

### Usage: get_gc_content.pl <fasta file> <output_file> ###

### This program takes a fasta file as it's input ###

### ###

### It returns a tab delimited file (output_file): column 1 = header ID (everything between ">" ###

### and the first space in the header), and column 2 = gc content for the fasta entry. ###

### ###

### Jennifer Meneghin ###

### July 23, 2009 ###

### ###

### This script now works properly with sequences that contain spaces. ###

### September 20, 2010 ###

### ###

### This script now also returns the total nucleotide count, along with the number of of ###

### A's, G's, C's and T's for each fasta record. ###

### September 21, 2010 ###

####################################################################################################

#---------------------------------------------------------------------------------------------------------------------------

#Deal with passed parameters

#---------------------------------------------------------------------------------------------------------------------------

if ($#ARGV == -1) {

usage();

exit;

}

$fasta_file = $ARGV[0];

$out_file = $ARGV[1];

unless ( open(IN, "$fasta_file") ) {

print "Got a bad fasta file: $fasta_file\n\n";

exit;

}

unless ( open(OUT, ">$out_file") ) {

print "Couldn't create $out_file\n";

exit;

}

print "Parameters:\nfasta file = $fasta_file\noutput file = $out_file\n\n";

#---------------------------------------------------------------------------------------------------------------------------

#The main event

#---------------------------------------------------------------------------------------------------------------------------

print OUT "ID\t% GCContent\tTotal Count\tG Count\tC Count\tA Count\tT Count\n";

$seq = "";

while (<IN>) {

chomp;

if (/^>/) {

#finish up previous line.

if (length($seq) > 0) {

&process_it;

}

#start new line.

$id = $_;

$id =~ s/^>(.+?)\s.+$/$1/g;

print OUT "$id\t";

}

else {

$seq = $seq . $_;

}

}

#finish up last line.

&process_it;

close(IN);

close(OUT);

sub usage {

print "Get GC Content\n";

print "Usage: get_gc_content.pl <fasta file>\n";

print "This program takes a fasta file as it's first (and only) parameter.\n\n";

print "It returns a tab delimited file (gc_out.txt): column 1 = header ID (everything between \">\"\n";

print "and the first space in the header), and column 2 = gc content for the fasta entry.\n\n";

print "Jennifer Meneghin\n";

print "July 23, 2009\n\n";

print "Updated September 20, 2010:\n";

print "This script now works properly with sequences that contain spaces.\n\n";

print "Updated September 21, 2010:\n";

print "This script now also returns the total nucleotide count, along with the number of of A's, G's, C's and T's for each fasta record.\n\n";

}

sub process_it {

@letters = split(//, $seq);

$gccount = 0;

$totalcount = 0;

$acount = 0;

$tcount = 0;

$gcount = 0;

$ccount = 0;

foreach $i (@letters) {

if (lc($i) =~ /[a-z]/) {

$totalcount++;

}

if (lc($i) eq "g" || lc($i) eq "c") {

$gccount++;

}

if (lc($i) eq "a") {

$acount++;

}

if (lc($i) eq "t") {

$tcount++;

}

if (lc($i) eq "g") {

$gcount++;

}

if (lc($i) eq "c") {

$ccount++;

}

}

if ($totalcount > 0) {

$gccontent = (100 * $gccount) / $totalcount;

}

else {

$gccontent = 0;

}

print OUT "$gccontent\t$totalcount\t$gcount\t$ccount\t$acount\t$tcount\n";

$seq = "";

}
