# Supplementary material for: Read Mapping and Transcript Assembly: A Scalable and High-Throughput Workflow for the Processing and Analysis of Ribonucleic Acid Sequencing Data
Source: Front Genet. 2020 Jan 24;10:1361. doi: 10.3389/fgene.2019.01361 (PMC6993073; doi:10.3389/fgene.2019.01361)
Supplement: File S3 — R code necessary to recapitulate visualization of data. [file Table_3.docx]

# Email authors at andrewnelson@email.arizona.edu for additional info

# Save this file as an R script and run in RStudio or your favorite R environment

# Import data from featureCounts

countdata <- read.table("feature_counts.csv", header=TRUE, row.names=1)

# Remove .bam or .sam from filenames

colnames(countdata) <- gsub("\\.[sb]am$", "", colnames(countdata))

# Convert to matrix

countdata <- as.matrix(countdata)

head(countdata)

# Assign condition

(condition <- factor(c(rep("Root", 60), rep("Flower", 35))))

# Analysis with DESeq2 ----------------------------------------------------

library(DESeq2)

# Create a coldata frame and instantiate the DESeqDataSet.

(coldata <- data.frame(row.names=colnames(countdata), condition))

dds <- DESeqDataSetFromMatrix(countData=countdata, colData=coldata, design=~condition)

dds

# Run the DESeq pipeline

dds <- DESeq(dds)

# Plot dispersions

png("qc-dispersions.png", 1000, 1000, pointsize=20)

plotDispEsts(dds, main="Dispersion plot")

dev.off()

# variance stabilizing transformation

vsd <- varianceStabilizingTransformation(dds)

head(assay(vsd))

hist(assay(vsd))

# Colors for plots below

## Ugly:

## (mycols <- 1:length(unique(condition)))

## Use RColorBrewer, better

library(RColorBrewer)

(mycols <- brewer.pal(8, "Dark2")[1:length(unique(condition))])

# Principal components analysis

DESeq2::plotPCA(rld, intgroup="condition")
